# Retention of pregnant women living with HIV across health care levels in Sierra Leone

**DOI:** 10.5588/pha.25.0052

**Published:** 2026-03-06

**Authors:** I.S. Turay, A.J. Bah, T. Sesay, D. Nair, E. Foday, R. Samuels, F. Lansana, R. Zachariah, M. Mustapha, J.S. Kanu, B.D. Fofanah, F. Kanu, J.A. Koroma, M.S. Kanu, W.K. Lahai, M.A. Sesay, G.N. Kamara, I.F. Kamara, S. Lakoh

**Affiliations:** 1 Ministry of Health, Government of Sierra Leone, Freetown, Sierra Leone;; 2 College of Medicine & Allied Health Sciences, University of Sierra Leone, Freetown, Sierra Leone;; 3 Boston College, School of Social Work, Chestnut Hill, MA, USA;; 4 UNICEF, UNAIDS, CDC, WHO Special Programme for Research and Training in Tropical Diseases (TDR), Geneva, Switzerland;; 5 KYM Consultancy Limited, Freetown, Sierra Leone;; 6 National Public Health Agency, Government of Sierra Leone, Freetown, Sierra Leone;; 7 34 Military Hospital, Defense Medical Services Republic of Sierra Leone Armed Forces, Freetown, Sierra Leone.

**Keywords:** PWLHIV, eMTCT, antenatal care, postpartum care, retention

## Abstract

**SETTING:**

The study was conducted across three health facilities in Sierra Leone, Princess Christian Maternity Hospital (tertiary), Rokupa Government Hospital (secondary), and George Brook Community Health Centre (primary).

**OBJECTIVES:**

To assess retention rates and identify factors associated with continued engagement of pregnant women living with HIV (PWLHIV) in antenatal and postnatal care.

**METHODS:**

A retrospective cohort study using 2024 routinely collected antenatal and elimination of mother-to-child transmission (eMTCT) data. Retention during antenatal and postnatal care was retrospectively assessed. Data were analysed, applying Poisson regression models to estimate relative risks.

**RESULTS:**

Of 397 PWLHIV enrolled, 84 (21.1%) were retained in antenatal care and 79 (25.9%) in postnatal care. Higher antenatal retention was linked to being newly diagnosed with HIV (adjusted relative risk [aRR] = 7.67), having no formal education (aRR = 2.99), and older age. These factors also predicted postnatal retention, with adjusted relative risks of 14.30 for newly diagnosed women, 1.34 for older women, and 2.96 for those with no formal education.

**CONCLUSION:**

Retention of pregnant and postpartum women living with HIV is low. Newly diagnosed, older, and less-educated women have better retention, while younger and already aware women struggle with engagement. We recommend enhanced counselling and targeted re-engagement strategies.

HIV remains a major global public health problem, with an estimated 40.8 million cases in 2024.^1,2^ Women are disproportionately more affected by HIV than men with 53% of all infections reported among adults aged 15 years or older.^1^ Global transmission is ongoing, as 120,000 new HIV infections in children less than 15 years were reported in 2024.^1,2^ The major source of HIV infection among children is mother-to-child transmission (MTCT), underscoring this as a public health challenge, particularly in the African region.^3^ Infant and childhood HIV transmission from mothers living with HIV can occur during pregnancy, labour, delivery, or breastfeeding.^4^ However, effective elimination of mother-to-child transmission (eMTCT) programmes – including sustained antenatal care (ANC) retention among pregnant women receiving antiretroviral therapy (ART) – can reduce HIV transmission risk to below 2% in non-breastfeeding mothers and below 5% in breastfeeding populations.^3,4^ Notwithstanding, retention of HIV-positive pregnant women in ANC and eMTCT services remains a major public health challenge in sub-Saharan Africa.^1,5^ Despite significant progress in the scale-up of lifelong ART under the ‘Option B+’ strategy, loss to follow-up among pregnant women continues to undermine the effectiveness of eMTCT programmes.^6,7^ Retention throughout pregnancy is critical to ensuring optimal maternal health, sustained viral suppression, and elimination of vertical HIV transmission.^8,9,10^

In Sierra Leone, the national HIV prevalence is estimated at 1.7% among adults aged 15–49 years, with women disproportionately affected.^9,11^ The global maternal mortality rate remains among the highest, estimated at 717 per 100,000 live births, underscoring the vulnerability of women during pregnancy and childbirth.^9,10,12^ The Ministry of Health adopted the WHO-recommended eMTCT strategy, which provides lifelong ART to all HIV-positive pregnant and breastfeeding women regardless of CD4 count.^4,11,13^ However, programme reports continue to highlight challenges with patient tracking, documentation, and continuity of care across facility levels.^11^ Retention in ANC services may differ between the three tiers of Sierra Leone’s health system; tertiary, secondary, and primary care, each characterised by varying resource availability, staff capacity, and patient loads. Understanding retention patterns and their determinants across these levels is essential for targeted programme improvements and the attainment of national eMTCT goals.

This study aimed to evaluate retention among HIV-positive pregnant women in antenatal and postnatal care by 1) determining retention rates and 2) identifying the factors that influence sustained engagement in ANC and postpartum services across all levels of the health care system in Sierra Leone.

## METHODS

A retrospective cohort study was conducted using routinely collected health facility records. Pregnant women living with HIV were identified at ANC registration, and retention outcomes were retrospectively ascertained from antenatal, eMTCT, and mother and neonate registers. All outcomes were determined solely from existing records; no participants were contacted or followed prospectively.

### General setting

Sierra Leone, a West African country with an estimated population of over 8 million, is administratively divided into five regions and 16 districts.^12^ Its health system is structured into primary, secondary, and tertiary levels of care.^12^ The primary level of care includes maternal and child health posts, community health posts, and community health centres. Secondary health care facilities include mainly district hospitals. Tertiary facilities are teaching or specialised hospitals.^12^

### Specific setting

The study was conducted in Freetown, the capital of Sierra Leone, across three facilities representing the three levels of health care delivery: The Princess Christian Maternity Hospital (PCMH) – tertiary level: PCMH is the national maternity referral hospital providing specialised obstetric services. It manages high-risk pregnancies and serves as the central site for providing eMTCT, ART services, viral load testing, and early infant testing. However, it is often overcrowded, leading to long waiting times and potential follow-up challenges. Rokupa Government Hospital (RGH) – secondary level: Located in Eastern Freetown, RGH provides essential maternal health and eMTCT services, including HIV testing, antiretroviral treatment services, and ANC follow-up. It serves as a referral point for surrounding Peripheral Health Units (PHUs) and nearby communities and represents a middle-resource setting within the urban public health system. George Brook Community Health Centre (GBCHC) – primary level: Situated within a densely populated peri-urban area. GBCHC delivers routine ANC, HIV testing, and antiretroviral treatment services. It is among the few primary facilities offering CD4 testing services. Its proximity to low-income communities provides valuable insight into retention dynamics among socio-economically disadvantaged populations.

### Study population and participants

The study included pregnant women who tested positive for HIV, enrolled in ANC services and initiated ART between January and December 2024 at three purposely selected health facilities in Sierra Leone. Women were included if they had a confirmed HIV diagnosis (either known prior to pregnancy or newly diagnosed during ANC registration) and evidence that the women had initiated, commenced, or continued ART at the time of ANC enrolment. Women were excluded if they had been transferred out to another facility, had missing identifiers preventing data linkage across registers, or did not have documented pregnancy or delivery outcomes data. For postpartum retention analyses, only women with documented live births and complete follow-up data were included.

### Outcome definition

Retention was defined as continued engagement in HIV care among the retrospective cohort of pregnant women living with HIV identified at ANC registration. Antenatal retention was defined as attendance of at least four ANC visits during pregnancy, in line with WHO recommendations. Postnatal retention was defined as documentation of at least one postnatal clinic visit within 6 weeks following delivery among women with a recorded live birth. Retention outcomes were assessed based on documented visits in routine facility registers.

### Data sources and variables

Data were extracted from the eMTCT register and the Mother and Neonate Register at each study facility. Extracted variables covered:

- Socio-demographic characteristics: age, residence, marital status, and education level
- Clinical and obstetric information: gravidity, HIV diagnosis status (known or newly diagnosed), number of ANC visits, delivery location and outcome, and postpartum follow-up visits

These variables were selected to enable assessment of retention across the antenatal and postnatal care continuum.

### Data management and analysis

Data were entered into Epicollect5 and exported to STATA version 16 for analysis. Categorical variables were summarised using frequencies and percentages, while continuous variables were summarised using medians and interquartile ranges. Retention outcomes were binary and relatively common; therefore, relative risks (RRs) were estimated instead of odds ratios. Crude RRs with 95% confidence intervals (CIs) were estimated using univariate Poisson regression. Multivariable analysis employed a generalised linear model with Poisson distribution and robust standard errors to estimate adjusted relative risks, accounting for clustering at the facility level.^14^ A *P* value of ≤0.05 was considered statistically significant.

### Ethical statement

Ethical approval was obtained from the Sierra Leone Ethics and Scientific Review Committee (SLESRC No. 017/02/2025) and the Ethics Advisory Group of The Union (EAG/8/21). Permission to access facility-level data was granted by the Ministry of Health. As this study utilised secondary, anonymised data, informed consent was not required. All datasets were de-identified prior to analysis, and electronic files were stored securely on password-protected devices.

## RESULTS

Of the 397 pregnant women living with HIV identified at ANC registration and forming the retrospective cohort, 266 (67%) were enrolled at PCMH, 98 (25%) at RGH, and 33 (8%) at GBCHC. Overall, 195 (49.1%) were aged 25–34 years, 389 (98%) resided in urban areas, 272 (68.5%) were married, and 253 (63.2%) attained secondary education (Table 1).

**TABLE 1.** Socio-demographic characteristics of HIV-positive pregnant women registered for antenatal care services in three selected health facilities in Sierra Leone between January 2024 and December 2024 (*N* = 397).

Characteristics	*N* (%)^A^
Age categories (in years)	
17–24	162 (40.8)
25–34	195 (49.1)
35–44	29 (7.3)
≥45	1 (0.2)
Not recorded	10 (2.5)
Residence	
Urban	389 (98.0)
Rural	8 (2.0)
Marital status	
Married	272 (68.5)
Single	88 (22.2)
Widow	1 (0.2)
Not recorded	36 (9.1)
Education	
Tertiary	10 (2.5)
Secondary	251 (63.2)
Primary	60 (15.1)
No formal education	2 (0.5)
Not recorded	74 (18.6)

*N* = total population.

A (%) = row percentage.

### Clinical characteristics and retention in care of pregnant women living with HIV

Retention outcomes were assessed within this retrospective cohort based on documented follow-up visits during pregnancy and the postnatal period. A total of 199 (50.1%) of the 397 women were newly diagnosed with HIV during ANC. Live birth at facility level was 305 (76.8%), and 293 (96.1%) attended at least one postnatal visit within 6 weeks of delivery (Table 2). Of the 397 pregnant women with HIV, 84 (21.1%, 95% CI: 17.2%–25.5%) were retained in care during the antenatal period, and 79 (25.9%, 95% CI: 21.1%–31.2%) were retained in care during the postnatal period (Table 2).

**TABLE 2.** Clinical characteristics and retention in care of pregnant women with HIV registered for antenatal care (ANC) services in three selected health facilities in Sierra Leone.

Characteristics	*N* (%)^A^
HIV diagnosis	
Known HIV-positive	198 (49.9)
Newly diagnosed during ANC visit	199 (50.1)
Gravidity	
Primigravida	17 (4.3)
Multigravida	156 (39.3)
Not recorded	224 (56.4)
Number of ANC visits	
4 or less	339 (85.4)
More than 4	58 (14.6)
Place of delivery	
Peripheral health unit	10 (2.5)
Hospital	298 (75.1)
Community	24 (6.0)
Not recorded	65 (16.4)
Delivery outcome	
Live birth	305 (76.8)
Still birth	24 (6.0)
Miscarriage	9 (2.3)
Not recorded	59 (14.9)
Postpartum care visits to health facility	
At least one visit within 6 weeks of delivery	293 (96.1)
No visits within 6 weeks	3 (0.98)
Data not available	9 (2.95)

*N* = total population.

A (%) = row percentage.

### Factors associated with retention in ANC

In adjusted analysis, women with no formal education had the highest retention (adjusted relative risk [aRR] = 2.99, 95% CI: 2.77–3.23, *P* < 0.001). Women newly diagnosed with HIV were more likely to be retained in care (aRR = 7.67, 95% CI: 2.69–21.86, *P* < 0.001) compared to those already known HIV-positive. Similarly, multigravida women had higher retention, while those with unrecorded gravidity showed significantly lower retention (aRR = 0.43, *P* < 0.001) (Table 3).

**TABLE 3.** Factors associated with retention in antenatal care among HIV-positive pregnant women registered for antenatal care services in three selected health facilities in Sierra Leone between January 2024 and December 2024 (*N* = 332).

Characteristics	Total	Retained in care	Unadjusted	Adjusted	*P* value
	*N*	*n* (%)^A^	RR^B^ (95% CI)	RR^B^ (95% CI)	
Age categories (in years)					
17–24	140	41 (29.3)	1.52 (1.46–1.58)	1.16 (0.93–1.45)	0.172
25–34	161	31 (19.3)	Ref	Ref	
35–49	24	10 (41.7)	2.16 (1.86–2.51)	1.81 (1.78–1.86)	<0.001
Not recorded	7	2 (28.6)	1.48 (1.38–1.59)	0.79 (0.52–1.20)	0.277
Marital status					
Single	70	17 (24.3)	Ref	Ref	
Married	236	64 (27.1)	1.11 (0.81–1.53)	1.11 (0.96–1.49)	0.115
Widow	1	0	—	—	
Not recorded	25	3 (12.0)	0.49 (0.16–1.48)	0.70 (0.17–2.87)	0.631
Education					
No formal education	2	1 (50.0)	1.96 (1.73–2.22)	2.99 (2.77–3.23)	<0.001
Primary	50	13 (26.0)	1.02 (0.77–1.36)	0.84 (0.49–1.46)	0.549
Secondary	228	58 (25.4)	Ref	Ref	
Tertiary	8	3 (37.5)	1.47 (0.62–3.51)	0.75 (0.36–1.54)	0.429
Not recorded	44	9 (20.5)	0.80 (0.71–0.91)	0.77 (0.72–0.82)	<0.001
HIV diagnosis					
Known HIV-positive	146	4 (2.7)	Ref	Ref	
Newly diagnosed	186	80 (43.0)	15.70 (5.79–42.52)	7.67 (2.69–21.86)	<0.001
Gravidity					
Primigravida	17	8 (47.1)	1.03 (0.63–1.69)	0.96 (0.53–1.72)	0.889
Multigravida	143	65 (45.5)	Ref	Ref	
Not recorded	172	11 (6.4)	0.14 (0.08–0.25)	0.43 (0.35–0. 52)	<0.001

CI = confidence interval.

A (%) = column percentage of antenatal care.

B RR = adjusted and unadjusted risk ratio.

### Factors associated with retention in postnatal care following a live birth

Older women (35–49 years) (aRR = 1.34, 95% CI: 1.12–1.62, *P* = 0.001) and women with no formal education (aRR = 2.96, *P* < 0.001) were highly retained in postnatal care. Women newly diagnosed with HIV had much higher postnatal retention (aRR = 14.30, 95% CI: 5.39–37.90, *P* < 0.001) than women who already knew their status. Marital status showed borderline significance, with married women tending to have higher retention (aRR = 1.22, *P* = 0.064). (Table 4).

**TABLE 4.** Factors associated with retention in postpartum care following a live birth among HIV-positive pregnant women registered for antenatal care services in three selected health facilities in Sierra Leone between January 2024 and December 2024 (*N* = 295).

Characteristics	Total *N*	Retained in care	Unadjusted	Adjusted	*P* value
		*n* (%)^A^	RR^B^ (95% CI)	RR^B^ (95% CI)	
Age categories (in years)					
17–24	132	40 (30.3)	1.43 (1.17–1.77)	1.21 (0.78–1.87)	0.402
25–34	133	28 (21.1)	Ref	Ref	
35–49	23	9 (39.1)	1.86 (1.27–2.71)	1.34 (1.12–1.62)	0.001
Not recorded	7	2 (28.6)	1.36 (1.27–1.45)	0.85 (0.49–1.47)	0.565
Residence					
Urban	290	79 (27.2)	—	—	
Rural	5	0			
Marital status					
Single	63	16 (25.4)	Ref	Ref	
Married	212	60 (28.3)	1.11 (0.99–1.26)	1.22 (0.99–1.52)	0.064
Widow	1	0	—	—	
Not recorded	19	3 (15.8)	0.62 (0.25–1.55)	0.88 (0.26–2.96)	0.839
Education					
No formal education	2	1 (50.0)	1.87 (1.75–2.01)	2.96 (2.61–3.36)	<0.001
Primary	46	12 (26.1)	0.98 (0.72–1.33)	0.78 (0.39–1.55)	0.478
Secondary	206	55 (26.7)	Ref	Ref	
Tertiary	6	2 (33.3)	1.25 (0.71–2.19)	0.67 (0.43–1.03)	0.066
Not recorded	35	9 (25.7)	0.96 (0.90–1.03)	0.79 (0.75–0.84)	<0.001
HIV diagnosis					
Known HIV-positive	128	4 (3.1)	Ref	Ref	<0.001
Newly diagnosed	167	75 (44.9)	14.37 (5.14–40.14)	14.30 (5.39–37.90)	

CI = confidence interval.

A (%) = column percentage of postnatal care.

B RR = adjusted and unadjusted risk ratio.

## DISCUSSION

The eMTCT programme in Sierra Leone started in 2004 and has been scaled up to achieve national coverage of 87%,^15^ but our study reports persistent programmatic gaps, with only 21.1% and 25.9% of pregnant women living with HIV retained in care during the antenatal and postnatal periods, respectively. Retention in care is influenced by several patient-level factors such as gravidity, level of education, age, marital status, and status of HIV diagnosis.

The WHO recommends at least four ANC visits to effectively monitor foetal and maternal wellbeing, treat crucial infections such as HIV, and offer guidance on newborn care.^13^ Our study applied this standard to define retention in antenatal and postnatal care, but the results were disappointingly low. Our findings align with studies from Cameroon, Uganda, and Malawi, which reported low ART uptake and early retention challenges among HIV-positive pregnant women in informal or lower-level health facilities.^8,16-18^ A higher retention is reported among 65.5% of pregnant women 3 months post-initiation of ART in Cameroon.^19^ In Indonesia, the reported retention rate among pregnant women with HIV was 67% 6 weeks post-initiation of ART. While the difference in retention across Africa and Asia may reflect gaps in programme implementation, it may be explained by variations in the definition of retention. Unlike other studies, which used duration to define retention, our study utilised ANC coverage as an indicator of access and use of health services during pregnancy. Despite this, a recent preprint article highlighted gaps in the initiation of ART and facility delivery of pregnant women living with HIV in Sierra Leone,^20^ emphasising the need to improve retention in both antenatal and postnatal care by pairing pregnant women with mentor mothers and urban community health workers. The MoMent study in Nigeria demonstrated the feasibility and impact of structured Mentor Mother interventions on retention and PMTCT outcomes.^19,21^ Furthermore, the higher universal ANC coverage of 78.2% reported in the 2019 demographic health survey in Sierra Leone emphasise the need for early ANC initiation among pregnant women living with HIV. Community support groups of people living with HIV should embed antenatal and postnatal education in their treatment literacy programmes to enhance early ANC attendance.

The proportion of documented facility-based live birth delivery in our study of 76.8% is lower than the reported over 83% of facility deliveries resulting in missed opportunities for maternal and infant interventions, including early initiation of zidovudine and nevirapine prophylaxis for HIV exposed infants, thereby increasing the risk of MTCT of HIV. The high postnatal retention rate of 96% among women with live births is encouraging, but it may be driven by infant vaccination at 6 weeks as the data mirror pentavalent vaccine uptake of 96% at 6 weeks of birth. This encouraging trend should be explored for integration of early infant HIV testing and treatment initiation where necessary.^22,23^ Nonetheless, missing data on postpartum visits and infant HIV outcomes (over 60% unrecorded) pose challenges for programme monitoring and evaluation. Challenges with documentation and register completeness may have contributed to underreporting facility-based deliveries and postpartum retention.^24,25^

Several patient-level characteristics have a positive influence on pregnant women’s retention in antenatal and postnatal care, emphasising the need for patient-centred, contextual, and pragmatic differentiated service delivery models. Differentiated service delivery models have been shown to improve retention and patient satisfaction by tailoring HIV services to individual needs and facility contexts.^26^ Multigravida and less-educated women were more likely to be retained in antenatal and postnatal care. One of the established approaches employed by the Sierra Leone eMTCT programme is the use of mentor mothers in following up pregnant women and HIV-exposed infants until the final infant outcome.^6,27^ The focused support provided to less-educated women through counselling, treatment literacy, and other forms of psychosocial support services by mentor mothers could explain these findings. Other socio-demographic analyses highlighted that age influences retention. Older women were more likely to stay engaged, possibly due to greater perceived risk, prior maternal experience, or community support mechanisms. Evidence from maternal intervention studies highlights the importance of integrated care, early ART initiation, and psychosocial support in improving PMTCT outcomes.^28^ The finding that newly diagnosed HIV-positive women had significantly higher retention during both ANC and postpartum periods suggests that active follow-up and counselling immediately after diagnosis are highly effective retention strategies, though challenges persist in early ANC engagement.^29^ Given the persistent HIV burden and TB co-infection risks, retention in care remains a critical priority.^30^

This study is the first to provide programmatic evidence on retention among pregnant women with HIV across the different levels of care in Sierra Leone, which stands out as a major strength of the study. However, incompleteness of data on gravidity and postpartum records may have compromised analysis of the variables.

## CONCLUSION

Our study shows low overall retention of pregnant and postpartum women living with HIV in antenatal and postnatal care. Retention was higher among newly diagnosed women, older women, and those with no formal education, while younger women and those already aware of their HIV status demonstrated poorer engagement. These findings highlight the need for targeted counselling, treatment literacy, and re-engagement strategies, particularly for known HIV-positive and younger women – to strengthen early ANC initiation and continuity of care.
